# Successful use of pessary for uterine prolapse after pelvic trauma in a nulliparous young female: A case report

**DOI:** 10.1097/MD.0000000000010139

**Published:** 2018-03-23

**Authors:** Fang-Fang Ai, Meng Mao, Ye Zhang, Jia Kang, Lan Zhu

**Affiliations:** Department of Obstetrics and Gynecology, Peking Union Medical College Hospital, Peking Union Medical College, Chinese Academy of Medical Sciences, Beijing, P.R. China.

**Keywords:** pelvic organ prolapse, pelvic trauma, pessary

## Abstract

**Rationale:**

To date, sporadic studies have shown that a relationship exists between delayed pelvic organ prolapse (POP) and pelvic trauma, and these cases have all been managed with surgical procedures.

**Patient concerns:**

A 29-year-old, nulliparous (Gravida 0) woman without sexual experience was referred to our gynecology outpatient clinic, complaining of a protruding vaginal mass 5 years after a traffic accident (hit by a truck when she was walking) that caused serious multiple injuries.

**Diagnoses:**

Stage 2 cystocele, stage 3 uterine prolapse, and stage 2 rectocele, pelvic trauma history.

**Interventions:**

The woman was successfully managed with the Gellhorn size 2 pessary use.

**Outcomes:**

The short-term effect of pessary use was significant improvement.

**Lessons:**

This is the first case report of the successful use of a pessary for POP after pelvic trauma in a nulliparous young female.

## 1 Introduction

Cases of pelvic organ prolapse (POP) in young nulliparous women who have suffered from high-energy pelvic trauma have only been reported sporadically.^[1–3]^ However, the management options for this unique condition are challenging. This report is the first to describe management comprising pessary use in a 29-year-old nulliparous woman with prolapse after severe pelvic trauma.

## 2 Case report

A 29-year-old, nulliparous (Gravida 0) woman without sexual experience was referred to our gynecology outpatient clinic, complaining of a protruding vaginal mass 5 years after a traffic accident (hit by a truck when she was walking) that caused serious multiple injuries. The concomitant urinary symptoms included urinary incontinence immediately after the accident. She also experienced bleeding caused by the friction of the mass while walking. Among other injuries, she had traumatic ventral hernia, pelvic fracture, pubic symphysis fracture and dislocation, right femoral shaft fracture, perineal laceration, closed abdominal injury, and sigmoid colon rupture. She had a total of 12 sequential operations, including right femoral shaft fracture reduction and internal fixation, sigmoidostomy, pelvic fracture internal fixation, perineum injury repair, and debridement surgery. Of these operations, the internal fixation surgery of the pelvic fractures ultimately failed. Therefore, she continues to have severe pubic symphysis dislocation and anterior pelvic ring bone defects. Two years after the accident, a surgery comprising sigmoidostomy apotheosis was successfully performed. Since then, she developed symptoms of weak defecation. The last operation for debridement surgery was performed in 2013, and she was then discharged. Since then, she began lower limb rehabilitation programs and recovered well. Now, she can stand and walk slowly with the aid of crutches.

At this clinical examination, she had a stage 2 cystocele, a stage 3 uterine prolapse, and a stage 2 rectocele (Aa = +1, Ba = +1, C = +3, gh = 6, pb = 4, TVL = 8, Ap = 0, Bp = 0, D = −4). Her body mass index was 22.04 kg/m^2^. Her gynecological examination was normal except for vulva deformation and loose vaginal mucosa. The pelvic floor muscles were almost unable to contract autonomously. The patient did not have a disease that could cause increased intra-abdominal pressure. A subjective evaluation with the Pelvic Floor Distress Inventory was performed and included the Pelvic Organ Prolapse Distress Inventory-6 (POPDI-6), Urinary Distress Inventory-6 (UDI-6), and Colon Rectal Anal Distress Inventory-8 (CRADI-8) scores, which were 41.75, 79.25 and 68.25, respectively.

**Figure 1:**
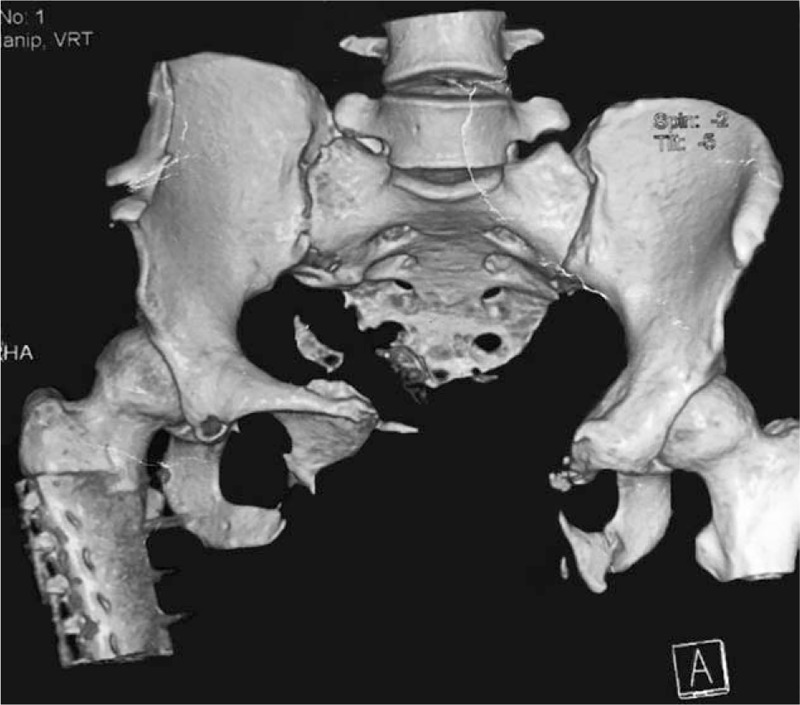
Computed tomography combined with 3-dimensional reconstruction shows the comminuted pelvic fracture, severe diastasis of the pubic symphysis, and the internal fixation change of the right femoral shaft fracture.

Computed tomography (CT) combined with 3-dimensional reconstruction showed the comminuted fracture of the right ala of the ilium and the bilateral of the body of the ilium, sacrococcyxlatera, pubic symphysis, pubic ramus, and ischial ramus, and also severe diastasis of the pubic symphysis and the internal fixation change of the right femoral shaft fracture (Fig. 1).

After proper counseling and discussion with the patient, conservative management with a pessary treatment was performed. At first, we attempted to place a ring with a support pessary (Cooper Surgical, Trumbull, CT), but ultimately failed as the pessary dropped out of the vagina when the patient stood up. Then, a Gellhorn pessary (Cooper Surgical, Trumbull, CT) was inserted, and she was asked to walk and perform Valsalva maneuvers with pessary placement. Afterward, she achieved prolapse reduction above the hymen successfully and did not feel any discomfort; so finally, the Gellhorn size 2 pessary was selected. She was taught how to manage the pessary, which included removing, replacing, and cleaning it regularly. We advised her to remove and clean the pessary for 1 night of no pessary rest at least once a week.

Two weeks after the initial fitting, she was asked to return for an assessment. The fitting was considered successful because she felt comfortable and wanted to continue to use the pessary. Then, at the 3-month follow-up visit, she felt very satisfied with the successful pessary use according to the Patient Global Impression of Change questionnaire for assessing satisfaction, although there was little improvement in voiding and defecation. There was no ulcer or other abnormality according to the vaginal examination, and there was no vaginal discharge, bleeding, pain, or other discomfort. The subjective evaluations of the POPDI-6, UDI-6, and CRADI-8 scores were 8.25, 70.75, and 68.75, respectively, which indicated obvious improvements in the prolapse symptoms.

Ethical approval was not necessary because this study was only a case report. Written informed consent was obtained from the patient.

## 3 Discussion

Studies have shown a wide range symptoms of pelvic floor dysfunction occur after pelvic trauma in women, and the most common symptoms are those affecting the bladder, the bowel, and sexual function.^[4]^ Although the etiology of POP is multifactorial, sporadic reports have shown that pelvic trauma may also be related to advanced POP. The underlying mechanisms may be due to bony pelvic disruption, injuries to the supports of the visceral fascia, neuropathies, and the muscular pelvic floor.^[5]^ In our case, the patient was a nulliparous, nonoverweight young woman with no predisposing factors who still progressed to symptomatic POP 5 years after the severe pelvic trauma. Thus, the prolapse herein seems to be directly related to the pelvic trauma and nerve injury.

Currently, because of the sporadic reports of pelvic trauma and POP, our understanding of the ideal treatment option is limited. The existing cases have shown that the treatment options for this unique condition are the same as those for the general population, and are based on individual findings.^[1–3,5]^ To date, we have retrieved a total of 4 studies on the treatment of delayed POP after pelvic trauma with disruption to the normal anatomy of the pelvis (Table 1). All of these case were managed with surgical treatments, of which autologous tissue repair was performed in 2 cases,^[1,5]^ anterior vaginal repair with a polypropylene mesh with sacrospinous ligament fixation of the uterus and posterior vaginal repair with perineorrhaphy was performed in 1 case,^[2]^ and abdominal wall cervicopexy was primarily performed in 1 case; the latter case showed rapid recurrence and was finally successfully managed by sacrospinous hysteropexy with a predesigned vaginal mesh along with anterior colporrhaphy and colpoperineorrhaphy.^[3]^

**Table 1:**
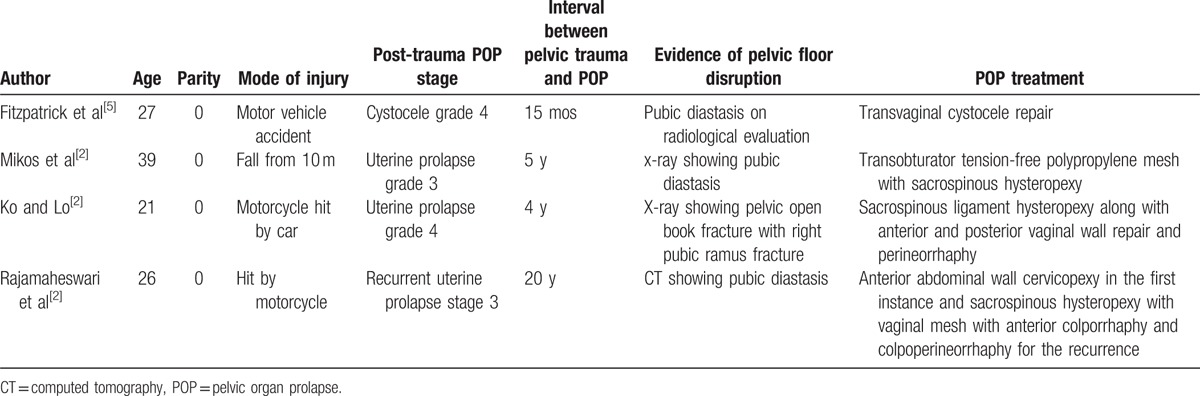
Review of the literature on the treatment of POP after pelvic trauma.

Pessary use as an excellent and effective conservative treatment for POP has received increasing attention. A survey conducted by the American Urogynecologic Society has shown that almost two-thirds of physicians choose a pessary over surgery as the first-line treatment for POP,^[6]^ and the pessary is especially suitable for women who have no surgical indications, who experience failed surgical treatment, who reject surgical treatment, or who have contraindications to surgery due to medical complications.

In our case, the patient was afraid of reoperation due to her experience of too many surgeries. In addition, the patient's pelvic ring was not stable enough because the internal fixation operation for pelvic fractures was not performed. However, for all of the cases in the literature, pelvic reconstructive surgery was performed after the orthopedic stabilization of the patients. Moreover, the patient in our case also had a desire to be treated with conservative therapy because of her fear of surgery. Therefore, we preferred to perform pessary treatment initially. In the end, the short-term effectiveness obtained after 3 months of pessary use was promising for relieving prolapse symptoms. However, a longer follow-up time is required to validate the long-term effectiveness of pessary treatment.

## 4 Conclusions

In conclusion, although rarely observed, pelvic trauma is a cause of POP in nulliparous young woman. This report is the first to highlight the feasibility and success of pessary use to manage the delayed onset of POP, where the normal pelvic anatomy is distorted.

## 5 Author contributions

**Data curation:** F-F. Ai.

**Investigation:** F-F. Ai, M. Mao, L. Zhu, Y. Zhang, J. Kang.

**Supervision:** L. Zhu.

**Writing – original draft:** F-F. Ai, L. Zhu.

**Writing – review & editing:** F-F. Ai, L. Zhu.
